# A radiomic model leveraging conventional and Hessian matrix-based radiomic features from DCE-MRI for predicting efficacy of neoadjuvant chemotherapy in patients with HER2-low breast cancer

**DOI:** 10.3389/fmed.2025.1639977

**Published:** 2026-01-13

**Authors:** Yuwei Zou, Bingxin Zhao, Yan Mao, Meng Lv, Yongmei Wang, Xiaohui Su, Zaixian Zhang, Jie Wu, Qi Wang

**Affiliations:** 1 Department of Pathology, The Affiliated Hospital of Qingdao University, Qingdao, Shandong, China; 2 Department of Radiation Oncology, The Affiliated Hospital of Qingdao University, Qingdao, Shandong, China; 3 Breast Disease Diagnosis and Treatment Center, The Affiliated Hospital of Qingdao University, Qingdao, Shandong, China; 4 Department of Radiology, The Affiliated Hospital of Qingdao University, Qingdao, Shandong, China

**Keywords:** HER2-low, breast cancer, DCE-MRI, radiomics, neoadjuvant chemotherapy

## Abstract

**Purpose:**

This study aimed to develop a predictive model for assessing the efficacy of neoadjuvant chemotherapy (NAC) in patients with Human Epidermal Growth Factor Receptor 2 (HER2)-low breast cancer, integrating clinical factors and radiomic features.

**Method:**

We retrospectively analyzed data from patients with HER2-low breast cancer who underwent NAC. Radiomic features were extracted from pre-treatment imaging, including wavelet-based and Hessian matrix-based features. Various machine learning models were constructed using radiomic and clinicopathological features. The Shapley additive explanations (SHAP) analysis was used to assess feature contributions. Model performance was evaluated based on the area under the receiver operating characteristic curve (AUC), accuracy, sensitivity, specificity, and other metrics. Finally, a nomogram was established by combining the best-performing models to enhance predictive utility.

**Results:**

For the clinicopathological model, the Random Forest (RF) algorithm was determined as the most effective. In the radiomic model, which incorporated both conventional and Hessian matrix-based features, RF exhibited superior performance compared to other models, achieving an AUC of 0.84 in the training cohort and 0.74 in the validation cohort. Based on these findings, a nomogram was developed that integrated the best-performing RF models from both the clinicopathological and radiomic feature sets. This nomogram attained an AUC of 0.89 in the training cohort and 0.79 in the validation cohort. Decision curve analysis further validated that the nomogram provided significant clinical benefits over models using only clinical or radiomic features.

**Conclusion:**

Our current study successfully constructed a predictive nomogram that offers a promising strategy for predicting NAC efficacy in patients with HER2-low breast cancer.

## Introduction

Breast cancer is one of the most prevalent malignancies worldwide, posing significant challenges in both diagnosis and therapy. In 2022 alone, there were 2.3 million new breast cancer diagnoses worldwide, resulting in approximately 665 thousand deaths (1). The Human Epidermal Growth Factor Receptor 2 (*HER2*) gene is a crucial biomarker in breast cancer, impacting classification, treatment decisions, and disease prognosis (2). Over the years, the identification of HER2-overexpression tumors has facilitated the development of targeted anti-HER2 therapies, which have substantially enhanced the treatment efficacy for HER2-positive breast cancer patients (3, 4). However, the current classification of HER2-negative cases into broad subtypes, including luminal or triple-negative breast cancer (TNBC), may not always be sufficiently precise or comprehensive, highlighting the need for more tailored treatment approaches.

Recent clinical trials have revealed that certain patients previously classified as HER2-negative, specifically those exhibiting low HER2 expression (defined as immunohistochemistry [IHC]1+ or IHC 2+ with negative fluorescence *in situ* hybridization (FISH) results, also known as IHC 2+/FISH–), could benefit from innovative anti-HER2 antibody-drug conjugates (ADCs) (5, 6). These ADCs, which couple targeted HER2 therapy with potent chemotherapy agents, have shown promise in improving outcomes for this subgroup of patients. However, there remains a paucity of data regarding the clinical features, chemotherapy responses, and prognostic factors for patients with HER2-low breast cancer, particularly those undergoing neoadjuvant chemotherapy (NAC). Neoadjuvant treatment is regarded as the conventional therapy for patients with locally advanced and early-stage invasive breast cancer, especially those who are candidates for breast-conserving surgery (7, 8). Approximately 30%−50% of patients with breast cancer following NAC achieve a pathological complete response (pCR) (9). However, this percentage is further reduced in HER2-low patients, with retrospective studies reporting that only 11.8%−16.4% of HER2-low patients achieve pCR after undergoing NAC (10–12).

Studies investigating the efficacy of NAC in patients with HER2-low breast cancer have yielded important insights. Liu et al. (13) conducted a study to investigate factors determining the efficacy of NAC in patients diagnosed with HER2-low triple-negative breast cancer. Their findings indicated that the NAC response in these patients was associated with Ki-67 expression, pathological grading, and changes in the HER2 status after neoadjuvant therapy. Another study enrolled a total of 905 HER2-negative patients who underwent at least four cycles of NAC (12). The results suggested that estrogen receptor (ER) status, lymph node staging, and Ki-67 represent potential factors influencing NAC efficacy in HER2-low patients. A retrospective study of 975 breast cancer patients with HER2-negative status following NAC assessed the pCR rate and prognosis for both the overall group and its subgroups (10). Within the stratified analysis of 579 HER2-low cases, it was observed that clinical T stage, N stage, ER status, and PR status significantly affected the pCR following NAC. Overall, current research exploring the effectiveness of NAC in the HER2-low population is based on retrospective analyses of clinical and pathological characteristics. Moreover, most studies that analyzed NAC efficacy in HER2-negative patients grouped them collectively, without separately investigating the HER2-low subgroup. However, existing research has shown that HER2-low and HER2-zero patients exhibit significant differences in clinical pathology characteristics, treatment response, and long-term prognosis, indicating they represent two distinct disease entities (10–13). Therefore, there is an urgent need to develop an effective predictive model to accurately assess the treatment response of HER2-low patients before NAC. Indeed, early prediction of treatment efficacy allows for better identification of HER2-low patients who will benefit from sole NAC. This not only helps optimize treatment decisions but also enables the customization of more personalized NAC regimens, including whether to introduce newer drugs or adopt more intensive combination chemotherapy during NAC.

Radiomics represents a field that aims to correlate imaging features with the biological characteristics of tumors, treatment response, and clinical outcomes (14, 15). The past few years have witnessed burgeoning interest in the use of radiomics for predicting NAC efficacy in breast cancer patients (16–18). However, studies on radiomic models aimed at forecasting the effectiveness of NAC in patients with HER2-low breast cancer remain limited.

The Hessian matrix is a key mathematical concept consisting of the second-order partial derivatives of a multivariable function, arranged in a square matrix. In radiomics, the Hessian matrix is used to characterize the local curvature characteristics of an image, with applications in areas such as vascular enhancement and feature point detection. Initially, Fusco et al. (19) developed a semiautomatic vascular mapping method for DCE-MRI using a Hessian matrix-based approach and morphological operators. Increased vascularity has been identified as a high-risk factor for breast cancer development. Xie et al. (20) combined peritumoral vascular and intratumoral radiomic features derived from the Hessian matrix to establish a radiomic model. This model demonstrated an area under the curve (AUC) of 0.82 in the training set and 0.67 in the internal validation set for predicting pCR. These results collectively indicate that the exploration of Hessian matrix-derived radiomic features in assessing the efficacy of NAC for breast cancer remains inadequately addressed in clinical research. Therefore, our study aimed to evaluate the performance of a radiomic model that incorporates both conventional and Hessian matrix-based features from DCE-MRI to predict the efficacy of NAC in HER2-low breast cancer patients.

## Methods and materials

### Patients

The study involved 203 individuals who had been diagnosed with breast cancer at the Affiliated Hospital of Qingdao University in Qingdao, China, between 2020 and 2024. Due to its retrospective design, informed consent was not required. The ethical committee of the hospital provided approval for the study, adhering to the principles outlined in the Declaration of Helsinki. Inclusion of participants was contingent upon the following criteria: (1) histopathological confirmation of primary invasive breast cancer; (2) surgical treatment after NAC; (3) HER2-low patients (HER2 IHC 1+ or HER2 IHC 2+/FISH–); (4) systemic chemotherapy prior to surgery; (5) sufficient DCE-MRI scans before NAC; (6) NAC regimens based on NCCN or CSCO guidelines; (7) availability of full medical records. Exclusion criteria included: (1) distant metastasis; (2) history of other cancers; (3) incomplete NAC before surgery; (4) incomplete clinical data; (5) the first MRI being conducted after the start of NAC or post-breast tumor biopsy.

### Pathological evaluation

The Miller-Payne grading system is widely recognized as a reliable method for pathological evaluation following NAC. Patient following NAC was classified into five distinct grades: Grade 1 (G1), which signifies minimal alterations in cancer cells with no overall reduction in cell numbers; Grade 2 (G2), which indicates a decrease of less than 30% while maintaining a high total cell count; Grade 3 (G3), denoting a moderate reduction of 30%−89% in cancer cell numbers; Grade 4 (G4), characterized by a substantial reduction of 90% or greater, with only isolated clusters of cells remaining; and Grade 5 (G5), which denotes the complete lack of malignancy at the primary tumor location. Individuals categorized as G1, G2, or G3 were considered as non-responders, whereas those classified as G4 or G5 were recognized as responders.

### Magnetic resonance acquisition protocol

The imaging protocol began with a T1-weighted image acquired prior to the administration of contrast, followed by eight post-contrast T1-weighted images, all incorporating fat saturation. Following the intravenous administration of a gadolinium-DTPA contrast agent at a dosage of 0.2 ml/kg, a 20 ml saline flush was delivered at an approximate rate of 2 ml/s. The initial series of post-contrast images was acquired 60 s after the gadolinium-DTPA injection, with seven subsequent scans. Comprehensive details regarding the imaging methodology have been outlined in the literature (21).

### Tumor segmentation

The N4 bias field correction algorithm (22) was used to preprocess the images, addressing inhomogeneities in the MR data. Regions of interest (ROIs) were carefully delineated on every slice of the DCE-MRI during the phase of maximum enhancement, as determined through the time-intensity curve. Two radiologists, each with 5 years of professional practice, performed this procedure. The agreement in ROI delineation between different radiologists and between the same radiologist was used to evaluate inter- and intra-observer reproducibility, respectively. To evaluate the reliability of feature extraction, intraclass correlation coefficients (ICCs) were computed, revealing a substantial degree of consistency. The ICC values surpassed 0.75 for assessments conducted by both different observers and the same observer.

### Feature extraction

The extraction of radiomic features was conducted utilizing the PyRadiomics Python package (version 3.7.12; https://pyradiomics.readthedocs.io) on the OnekeyAI platform. All available features implemented in PyRadiomics, including wavelet and Laplacian of Gaussian transformations, and Hessian-based radiomic features were obtained from the original images. Key parameters for Hessian matrix feature extraction: Gaussian filtering standard deviation σ ranges from 0.5 to 2.0 with a step of 0.5; a 3 × 3 neighborhood window is used for matrix feature calculation; quantitative indicators include eigenvalues, Anisotropy, Linearity, Planarity, and Sphericity.

### Radiomic model building

In our study, patients' response to NAC (responders vs. non-responders) was used as the classification label for the machine learning model. The dataset was split randomly into two training and validation subsets at a 7:3 ratio. The predictive models were developed using the training subset and the extracted radiomic features. An initial evaluation of these features was conducted employing the Mann–Whitney *U* test, establishing a significance threshold at *P* < 0.05. Subsequently, the Pearson correlation coefficient was applied to assess pairwise relationships among the radiomic features, leading to the exclusion of features with a correlation coefficient |*r*| greater than 0.9. For the training cohort, feature selection was performed using the Least Absolute Shrinkage and Selection Operator (LASSO) methodology, with alpha set to 1, and the maximum number of iterations at 1,000. Both logistic regression (LR), Naive Bayes, Random Forest (RF), and multi-layer perceptron (MLP) models were developed for classification. A fivefold cross-validation was conducted using the StratifiedKFold function from scikit-learn, which split the training cohort into five non-overlapping subsets. In each iteration, one subset was used as the validation set, while the remaining subsets were merged to form the training set. This method ensured proportional representation of each class in both the training and validation sets, aiding in the identification of optimal model hyperparameters. Optimize the model hyperparameters for LR (penalty=“1”, max_iter=100), Random Forest (n_estimators=5, max_depth=3, min_samples_split=4, random_state=0), and MLP (hidden_layer_sizes = (61, 128, 64, 32), max_iter=300, solver=“sgd”, random_state=0). Other parameters remained at a default level. Model performance was evaluated using Receiver Operating Characteristic (ROC) analysis, along with the calculation of sensitivity, specificity, positive predictive value (PPV), negative predictive value (NPV) and F1-score. Decision Curve Analysis (DCA) was implemented to evaluate the clinical relevance of our predictive models, thereby enhancing comprehension of the prospective advantages in a clinical setting. To demonstrate the concordance between predicted probabilities and actual outcomes, a calibration curve was also employed.

To evaluate the influence of each feature on the predictive results of the radiomic model, Shapley Additive Explanations (SHAP) values were applied. SHAP is a powerful tool for explaining machine learning model predictions, given that it quantifies the significance of each feature (23). Based on cooperative game theory, SHAP values provide a quantifiable assessment of the contribution of each feature to the predictions of a specific model, both in terms of magnitude and direction.

### Clinicopathological model development

Various clinical parameters, including histopathological characteristics, were evaluated for their potential impact on clinical deterioration. A clinical signature was developed, incorporating key factors such as the patient's age, ER status, PR status, and Ki-67 index. Subsequently, LR, Naive Bayes, Random Forest, and MLP models were employed to construct the classification models.

By integrating the most effective radiomic model with the clinicopathological model, a predictive nomogram was developed to improve clinical decision-making. The nomogram calculated a total score based on the contributions of both clinicopathological and radiomic signatures, which was then transformed into a linear predictor. This linear predictor served as a continuous value that reflects the overall prediction from both models. Finally, the linear predictor was used to calculate a risk score, which helped to categorize patients based on their likelihood of NAC efficacy. This integrated approach enabled a more personalized and accurate prediction by combining clinicopathological and radiomic signatures.

### Statistical analysis

Continuous variables were analyzed using either the *t*-test or the Mann–Whitney *U* test, contingent upon their statistical distribution. To evaluate categorical variables, χ^2^ tests were employed. We utilized R Studio (2023.12.1) and Python 3.12.2 for performing statistical analyses. Radiomic feature extraction was conducted using PyRadiomics version 3.7.12, while machine learning applications were implemented with Scikit-learn version 1.0.2. The construction of the ROC curve, calibration curve, DCA curve, and the Hosmer-Lemeshow test was facilitated by the “pROC,” “rms,” “rmda,” and “generalhoslem” packages. A significance threshold of 0.05 was set for all statistical analyses, using a two-tailed approach. The core code in the article can be found in Supplementary Data S1.

## Results

### Patient clinical features

**Figure 1:**
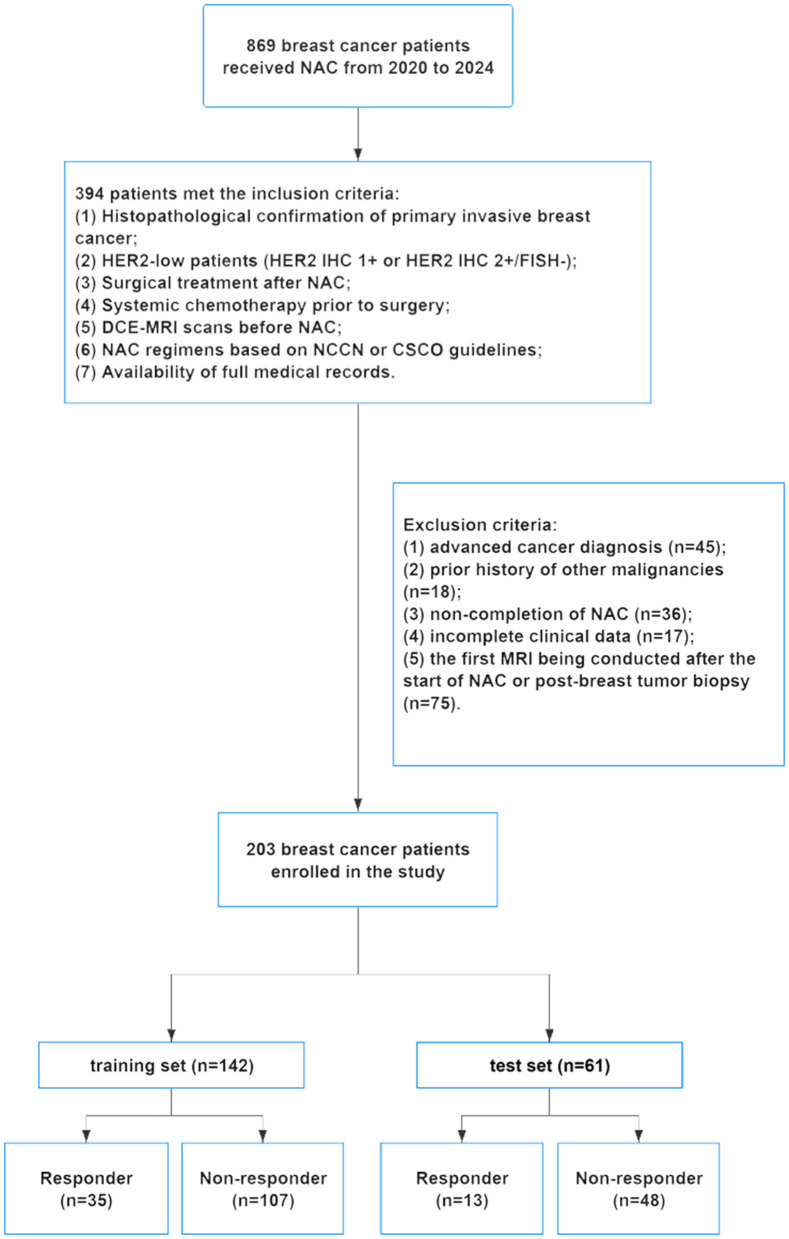
Flow chart of patient enrollment.

Between 2020 and 2024, a total of 869 DCE-MRI scans were initially collected. From this cohort, 394 patients satisfied the pre-defined inclusion criteria, while 191 patients were excluded for the following reasons: advanced cancer diagnosis (*n* = 45), prior history of other malignancies (*n* = 18), non-completion of NAC (*n* = 36), incomplete clinical data (*n* = 17), or the first MRI being conducted after the start of NAC or post-breast tumor biopsy (*n* = 75). The screening process is illustrated in the flowchart shown in Figure 1.

In this study, a total of 203 patients were involved and divided into training (*n* = 142) and validation (*n* = 61) cohorts. Table 1 outlines the baseline characteristics of these cohorts. Both cohorts exhibited comparable baseline characteristics, including hormone receptor status (ER-positive: 93.66 vs. 88.52%, *P* = 0.34; PR-positive: 76.76 vs. 80.33%, *P* = 0.58), and HER2 status (IHC 1+: 72.54 vs. 72.13%, *P* = 0.95). Other characteristics, such as clinical tumor stage and tumor-infiltrating lymphocytes (TILs), were also comparable. We further conducted stratified analyses of clinical characteristics between the training and validation cohorts, stratified by responder and non-responder. The results showed no statistically significant differences in major clinical characteristics between the two cohorts within each subgroup (see Supplementary Table S1). Therefore, the training and validation cohorts were well-matched for analyzing the efficacy of NAC in HER2-low breast cancer patients.

**Table 1:** The clinical characteristics of overall, training, and test cohort.

**Characteristics**	**Overall cohort**	**Training cohort**	**Validation cohort**	*P* value^*^
*n*	203	142	61	
Age, Mean ± SD	50.77 ± 9.77	50.19 ± 9.90	52.13 ± 9.39	0.20
**Menopausal status**, ***n*** **(%)**				
Pre-menopause	109 (53.69%)	79 (55.63%)	30 (49.18%)	0.40
Post-menopause	94 (46.31%)	63 (44.37%)	31 (50.82%)	
ER status, *n* (%)
Positive	187 (92.12%)	133 (93.66%)	54 (88.52%)	0.34
Negative	16 (7.88%)	9 (6.34%)	7 (11.48%)	
**PR**, ***n*** **(%)**				
Positive	158 (77.83%)	109 (76.76%)	49 (80.33%)	0.58
Negative	45 (22.17%)	33 (23.24%)	12 (19.63%)	
**Her-2**, ***n*** **(%)**				
IHC 1+	147 (72.41%)	103 (72.54%)	44 (72.13%)	0.95
IHC 2+/FISH-	56 (27.59%)	39 (27.46%)	17 (27.87%)	
Ki-67 index, median (IQR)	30 (20, 50)	30 (20, 50)	30 (20, 50)	0.50
**cT**, ***n*** **(%)**				
T1	20 (9.85%)	12 (8.45%)	8 (13.11%)	0.41
T2	97 (47.78%)	71 (50.00%)	28 (45.90%)	
T3	69 (33.99%)	48 (33.80%)	19 (31.15%)	
T4	17 (8.37%)	11 (7.75%)	6 (9.84%)	
**cN**, ***n*** **(%)**				
N0	17 (8.37%)	12 (8.45%)	5 (8.19%)	0.88
N1	157 (77.34%)	111 (78.17%)	46 (75.41%)	
N2	18 (8.87%)	12 (8.45%)	6 (9.84%)	
N3	11 (5.42%)	7 (4.93%)	4 (6.56%)	
ER value, median (IQR)	80 (60, 90)	80 (60, 90)	90 (70, 90)	0.43
PR value, median (IQR)	40 (5, 80)	35 (5, 80)	40 (10, 80)	0.58
TILs, median (IQR)	5 (3, 10)	5 (3, 10)	5 (5, 10)	0.17
**Efficacy**, ***n*** **(%)**				
Non-responder	155 (76.35%)	107 (75.4%)	48 (78.7%)	0.61
Responder	48 (23.65%)	35 (24.6%)	13 (21.3%)	

IQR, interquartile range.

^*^The P-value is for the comparison between the training and validation cohort.

### Clinicopathological factors related to treatment efficacy in HER2-low breast cancer

In both the training and validation cohorts, several clinicopathological factors were analyzed for their association with the efficacy of NAC in patients with HER2-low breast cancer (Table 2).

**Table 2:** The comparison of clinicopathological characteristics between responders and non-responders in training and validation cohort.

**Characteristics**	**Overall cohort**		***P* value**	**Training cohort**		***P* value**	**Validation cohort**		***P* value**
	**Responders**	**Non-responders**		**Responders**	**Non-responders**		**Responders**	**Non-responders**	
*n*	48	155		35	107		13	48	
Age, Mean ± SD	46.75 ± 8.35	52.02 ± 9.87	< 0.01	45.80 ± 8.27	51.64 ± 10.00	< 0.01	49.31 ± 8.35	52.89 ± 9.59	0.23
**Menopausal status**, ***n*** **(%)**									
Pre-menopause	36 (75.00%)	73 (35.96%)	< 0.01	28 (80.00%)	51 (47.66%)	< 0.01	8 (61.54%)	22 (45.83%)	0.32
Post-menopause	12 (25.00%)	82 (40.39%)		7 (20.00%)	56 (52.34%)		5 (38.46%)	26 (54.17%)	
**ER status**, ***n*** **(%)**									
Positive	41 (85.42%)	146 (94.19%)	0.04	30 (85.71%)	103 (96.26%)	0.07	11 (84.62%)	43 (89.58%)	0.99
Negative	7 (14.58%)	9 (5.81%)		5 (14.29%)	4 (3.74%)		2 (15.38%)	5 (10.42%)	
**PR**, ***n*** **(%)**									
Positive	30 (62.50%)	128 (82.58%)	< 0.01	22 (62.86%)	87 (81.31%)	0.03	8 (61.54%)	41 (85.42%)	0.13
Negative	18 (37.50%)	27 (17.42%)		13 (37.14%)	20 (18.69%)		5 (38.46%)	7 (14.58%)	
**Her-2**, ***n*** **(%)**									
IHC 1+	36 (75.00%)	111 (71.61%)	0.65	28 (80.00%)	75 (70.09%)	0.25	8 (61.54%)	36 (75.00%)	0.54
IHC 2+/FISH-	12 (25.00%)	44 (28.39%)		7 (20.00%)	32 (29.91%)		5 (38.46%)	12 (25.00%)	
Ki-67 index, median (IQR)	40 (30, 62.5)	30 (20, 42.5)	< 0.01	50 (30, 65)	30 (20, 40)	< 0.01	30 (30, 50)	30 (18.75, 50)	0.26
**cT**, ***n*** **(%)**									
T1	4 (8.33%)	16 (10.32%)	0.88	1 (2.86%)	11(10.28%)	0.61	3 (23.1%)	5 (10.41%)	0.14
T2	24 (50.00%)	73 (47.10%)		21 (60.00%)	50 (49.02%)		5 (38.5%)	23 (47.92%)	
T3	18 (37.50%)	51 (32.90%)		11 (31.43%)	37 (36.27%)		5 (38.5%)	14 (29.17%)	
T4	2 (4.17%)	15 (9.68%)		2 (5.71%)	9 (8.82%)		0 (0%)	6 (12.50%)	
**cN**, ***n*** **(%)**									
N0	4 (8.33%)	13 (8.39%)	0.75	4 (11.4%)	8 (7.48%)	0.66	0 (0%)	5 (10.42%)	0.47
N1	40 (83.33%)	117 (75.48%)		28 (80%)	83 (77.57%)		12 (92.31%)	34 (70.83%)	
N2	3 (6.25%)	15 (9.68%)		2 (5.7%)	10 (9.35%)		1 (7.69%)	5 (10.42%)	
N3	1 (2.08%)	10 (6.45%)		1 (2.9%)	6 (5.60%)		0 (0%)	4 (8.33%)	
ER value, median (IQR)	80 (5, 90)	90 (70, 90)	0.03	70 (4, 90)	80 (70, 90)	0.06	80 (5, 90)	90 (70, 90)	0.24
PR value, median (IQR)	10 (0, 50)	50 (5, 90)	< 0.01	30 (0, 70)	40 (5, 85)	0.04	10 (0, 30)	60 (10, 90)	< 0.01
TILs, median (IQR)	5 (3, 20)	5 (3, 10)	0.66	5 (3, 10)	5 (3, 10)	0.81	10 (8.25, 12.5)	5 (5, 10)	0.48

IQR, interquartile range.

In the training cohort, younger age, premenopausal status, PR positivity, and higher Ki-67 index were significantly associated with a better response to NAC. However, ER status, HER2 expression, and tumor stage showed no significant differences. In the validation cohort, the associations with treatment response were weaker. Premenopausal status and Ki-67 index did not demonstrate statistically significant differences, and factors like age, ER status, and HER2 expression were not significantly linked to NAC efficacy.

One possible explanation for the lack of a significant association in the validation cohort is its smaller sample size compared to the training cohort, which could reduce the statistical power to detect significant differences. Besides, inherent differences between the cohorts, such as slight variations in treatment protocols or patient characteristics, may contribute to these discrepancies.

### Development and validation of a clinicopathological signature

In the training cohort, univariate analysis identified several factors potentially related to the effectiveness of NAC in patients with HER2-low breast cancer. These factors included age, menopausal status, Ki-67 index, and ER and PR expression levels. Given that earlier research has suggested a link between HER2 expression levels and NAC efficacy, HER2 expression was also included as a potential predictor. The machine learning models were trained using these clinicopathological variables to predict NAC efficacy in HER2-low patients. The detailed performance metrics for each model, presented in Table 3, highlight the predictive power of the model built on these factors.

**Table 3:** The detailed results of machine learning models based on clinicopathological factors.

**Machine learning model**	**Accuracy**	**AUC**	**95% CI**	**Sensitivity**	**Specificity**	**PPV**	**NPV**	**F1-score**
**LR**								
Training cohort	0.68	0.80	0.72–0.87	0.91	0.61	0.43	0.96	0.34
Test cohort	0.54	0.60	0.42–0.79	0.77	0.48	0.29	0.89	0.13
**Naive Bayes**								
Training cohort	0.63	0.78	0.69–0.86	0.91	0.54	0.40	0.95	0.02
Test cohort	0.71	0.63	0.46–0.81	0.54	0.75	0.37	0.86	0.37
**Random Forest**								
Training cohort	0.81	0.84	0.78–0.91	0.77	0.82	0.59	0.92	0.55
Test cohort	0.79	0.65	0.45–0.84	0.46	0.88	0.50	0.86	0.53
**MLP**								
Training cohort	0.79	0.84	0.76–0.91	0.77	0.80	0.56	0.92	0.41
Test cohort	0.59	0.65	0.47–0.82	0.85	0.52	0.32	0.93	0.14

LR, logistic regression; MLP, multi-layer perceptron; AUC, area under curve; PPV, positive predictive value; NPV, negative predictive value.

The Random Forest model exhibited the best performance among the machine learning algorithms evaluated, reaching an accuracy of 0.81 and an AUC of 0.84 in the training dataset. Although its performance decreased on the validation set with 0.79 accuracy and an AUC of 0.65 (95% CI: 0.45–0.84), it still exhibited balanced PPV, and NPV. Other models, such as LR, Naive Bayes, and MLP, demonstrated satisfactory accuracy but did not outperform Random Forest, particularly in terms of validation set performance.

### Establishment and validation of a conventional and Hessian matrix-based radiomic model

**Figure 2:**
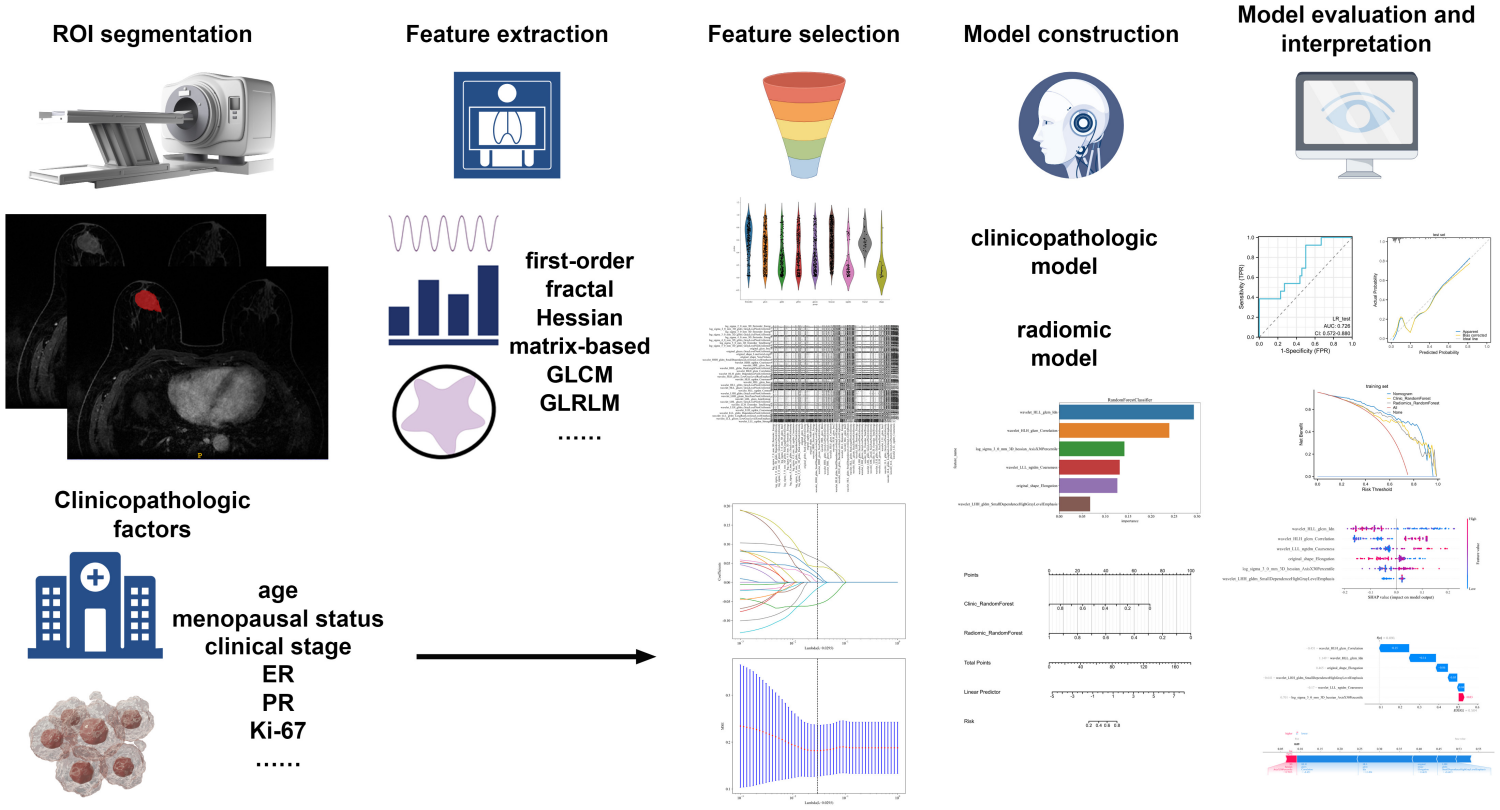
The flowchart of building conventional and Hessian matrix-based radiomic signature, clinical signature, and nomogram.

Figure 2 illustrates how machine learning models are built using conventional and Hessian matrix-based radiomic features, clinicopathological factors, and a nomogram. A total of 1,730 radiomic features were initially extracted. Using one-way ANOVA, 173 features were retained for further analysis. Following Pearson correlation testing, features with a correlation coefficient above 0.9 were filtered, leaving 21 features for the next stage. These 21 features were subjected to LASSO regression, which identified six features that were most relevant to predicting the efficacy of NAC.

**Figure 3:**
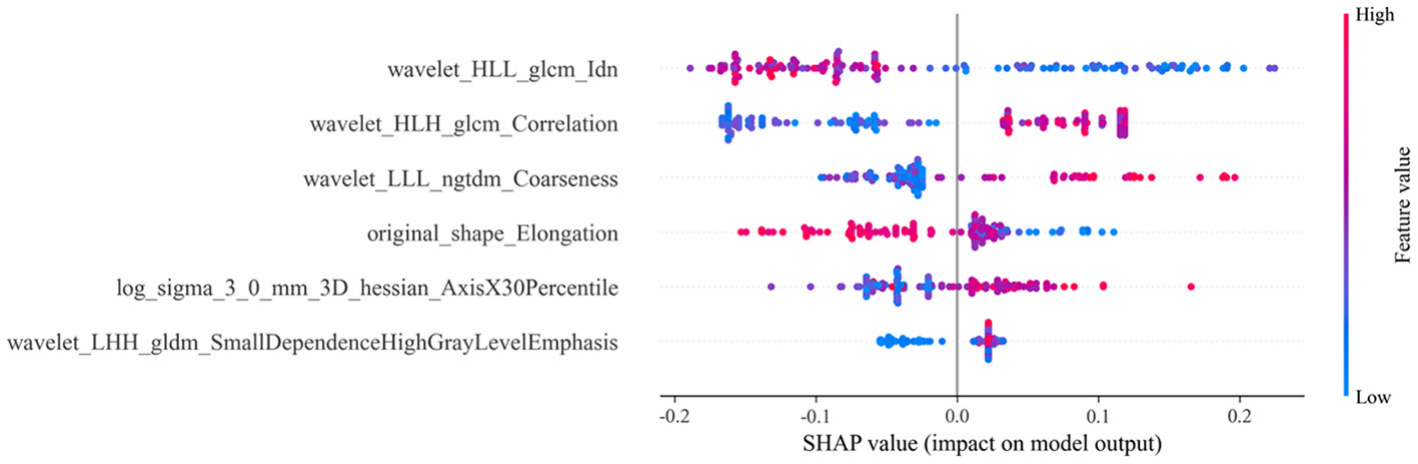
SHAP summary plot for conventional and Hessian matrix-based radiomic models. Each dot represents a patient, with colors indicating varying levels of influence on the model's output. SHAP stands for Shapley additive explanations; LALGLE, large area low gray level emphasis; DNUN, dependence non-uniformity normalized; ZE, zone entropy; SE, sum entropy; ZP, zone percentage; SAE, small area emphasis.

**Figure 4:**
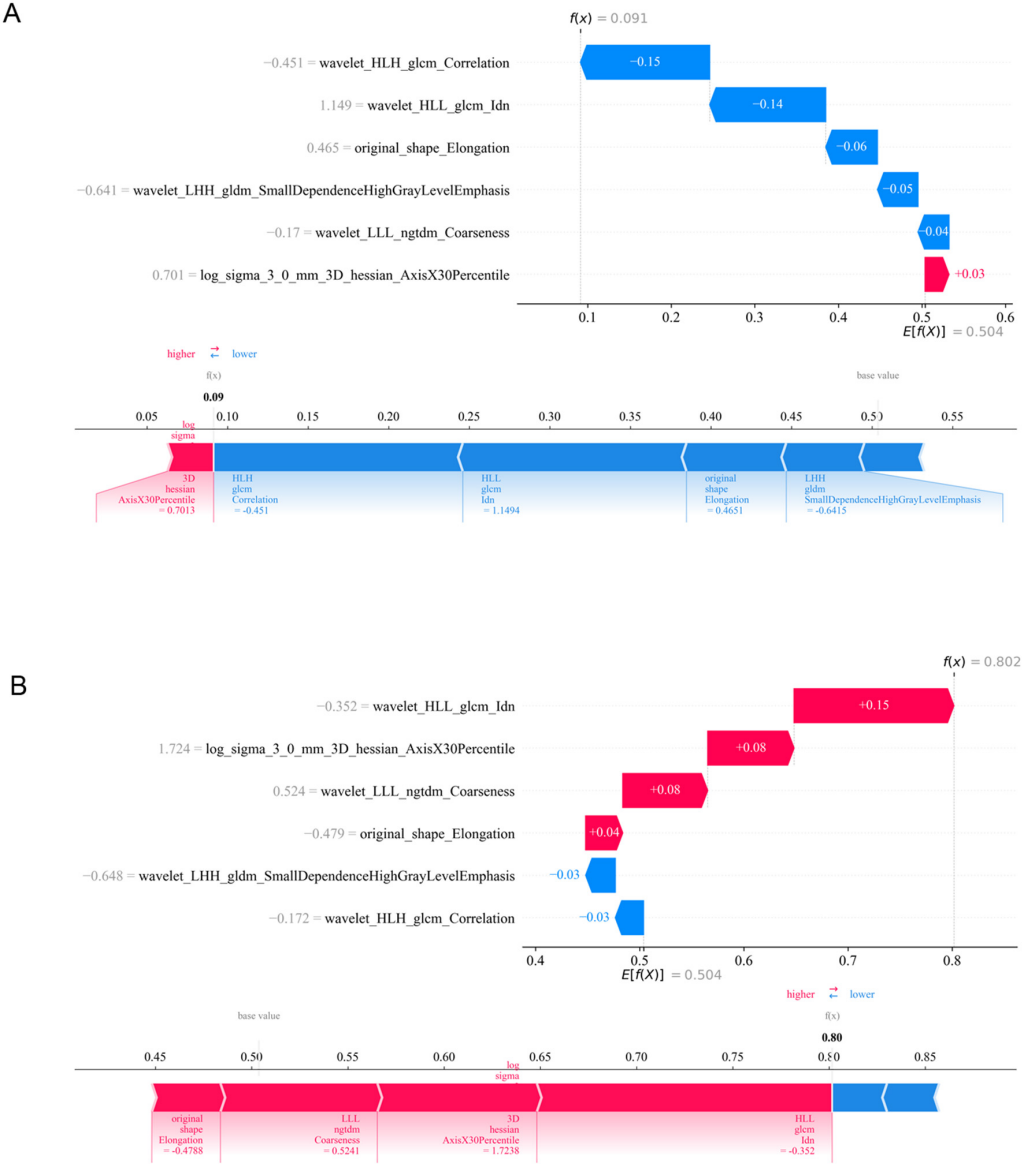
Application analysis of radiomic model for two representative HER2-low patients with varying responses to NAC. **(A)** HER2-low patient received G1–3 according to Miller-Payne grading system after NAC. **(B)** HER2-low patient received G4–5 according to Miller-Payne grading system after NAC. ZP, zone percentage; SAE, small area emphasis; ZE, zone entropy; LDHGLE, large dependence high gray level emphasis; DNUN, dependence non-uniformity normalized; ZV, zone variance.

These six features were used to construct the machine learning model aimed at predicting the efficacy of NAC in HER2-low breast cancer patients. To visualize the contributions of different features to the model, we used SHAP summary plots and scatter plots, revealing the distribution of SHAP values corresponding to each feature across the dataset (Figure 3). As depicted in the SHAP summary plot, the wavelet_HLL_glcm_Idn demonstrated the most significant impact on the model's contribution among the six radiomic features. The SHAP decision plot provides an explanation for the evaluation of individual patient cases. Figure 4 illustrates two representative HER2-low patients exhibiting differential responses to NAC.

**Figure 5:**
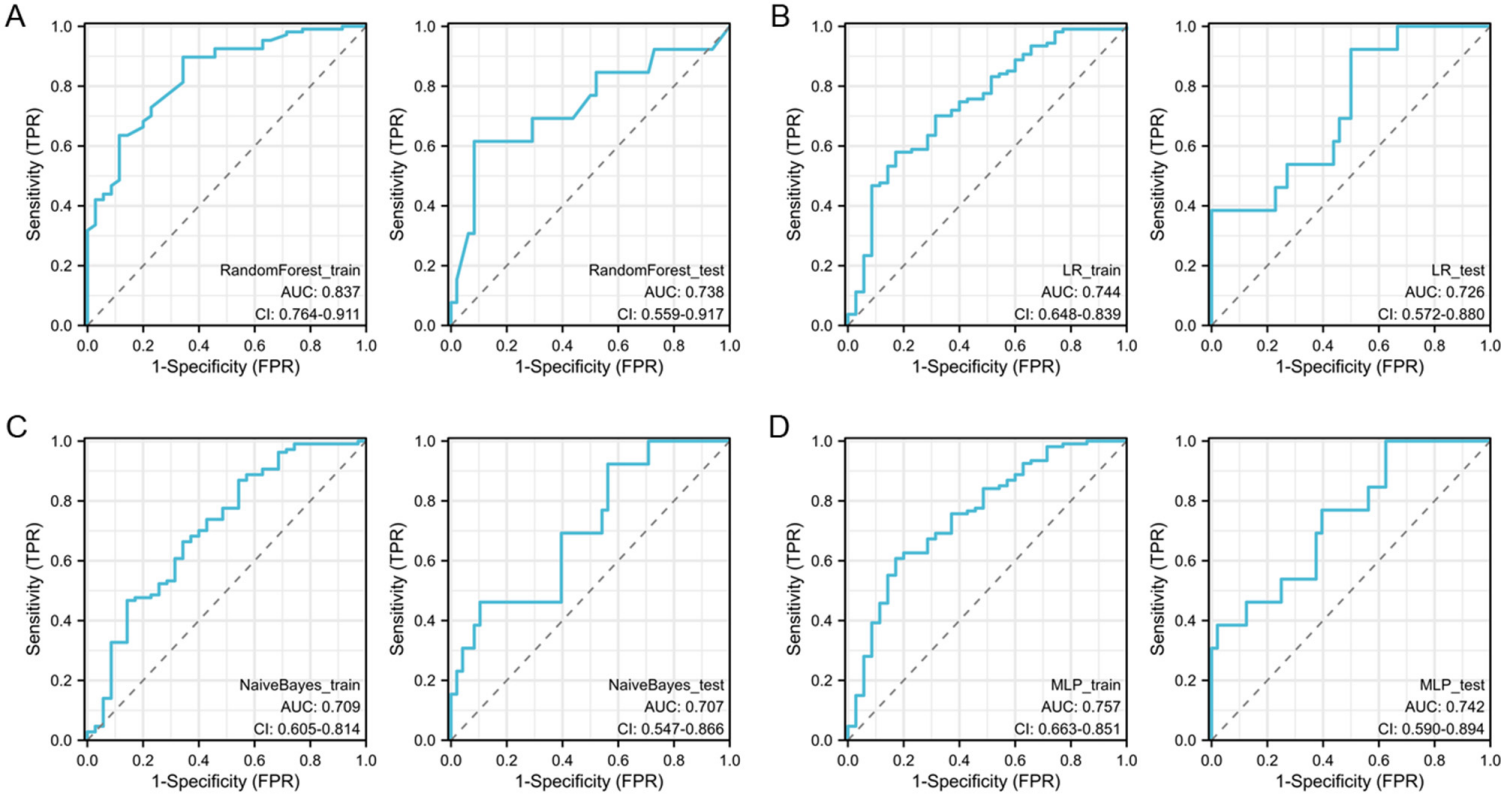
The ROC curves of different machine learning models for predicting efficacy of Her2-low breast cancer patients after NAC. **(A)** ROC curves for Random Forest model in the training and test cohort; **(B)** ROC curves for LR model in the training and test cohort; **(C)** ROC curves for Naive Bayes model in the training and test cohort; **(D)** ROC curves for MLP model in the training and test cohort.

The performance of machine learning models constructed using conventional and Hessian matrix-based radiomic features for forecasting the efficacy of NAC in patients with HER2-low breast cancer is summarized in Table 4. Random Forest exhibited the highest accuracy both in the training (0.84) and validation (0.85) sets, yielding AUC values of 0.84 and 0.74, respectively (Figure 5A). Furthermore, the model exhibited well-balanced sensitivity (0.62) and specificity (0.91) on the validation set. The PPV and NPV were 0.66 and 0.89, respectively, indicating that the model was effective in identifying patients likely to respond to NAC while maintaining strong reliability. The LR, Naive Bayes, and MLP models all exhibited various limitations, with poor accuracy or performance on the validation set (Figures 5B–D).

**Table 4:** The detailed results of machine learning models based on conventional and Hessian matrix-based radiomic features.

**Machine learning model**	**Accuracy**	**AUC**	**95% CI**	**Sensitivity**	**Specificity**	**PPV**	**NPV**	**F1-score**
**LR**								
Training cohort	0.64	0.74	0.65–0.84	0.83	0.58	0.39	0.91	0.53
Test cohort	0.59	0.73	0.57–0.88	0.92	0.50	0.33	0.96	0.51
**Naive Bayes**								
Training cohort	0.77	0.71	0.60–0.81	0.46	0.87	0.53	0.83	0.49
Test cohort	0.54	0.71	0.55–0.87	0.92	0.44	0.31	0.95	0.48
**Random Forest**								
Training cohort	0.84	0.84	0.76–0.91	0.66	0.90	0.68	0.89	0.67
Test cohort	0.85	0.74	0.46–0.92	0.62	0.91	0.66	0.89	0.50
**MLP**								
Training cohort	0.66	0.76	0.66–0.85	0.83	0.61	0.41	0.92	0.55
Test cohort	0.51	0.74	0.59–0.89	1.00	0.38	0.30	1.00	0.46

LR, logistic regression; MLP, multi-layer perceptron; AUC, area under curve; PPV, positive predictive value; NPV, negative predictive value.

It can be observed that the F1 scores of both the clinicopathological model and the radiomic model are not ideal. Therefore, we further combined the clinicopathological model and the radiomic model to construct a nomogram, which was evaluated as a tool for predicting the efficacy of NAC in patients with HER2-low breast cancer.

### Nomogram development and validation

**Figure 6:**
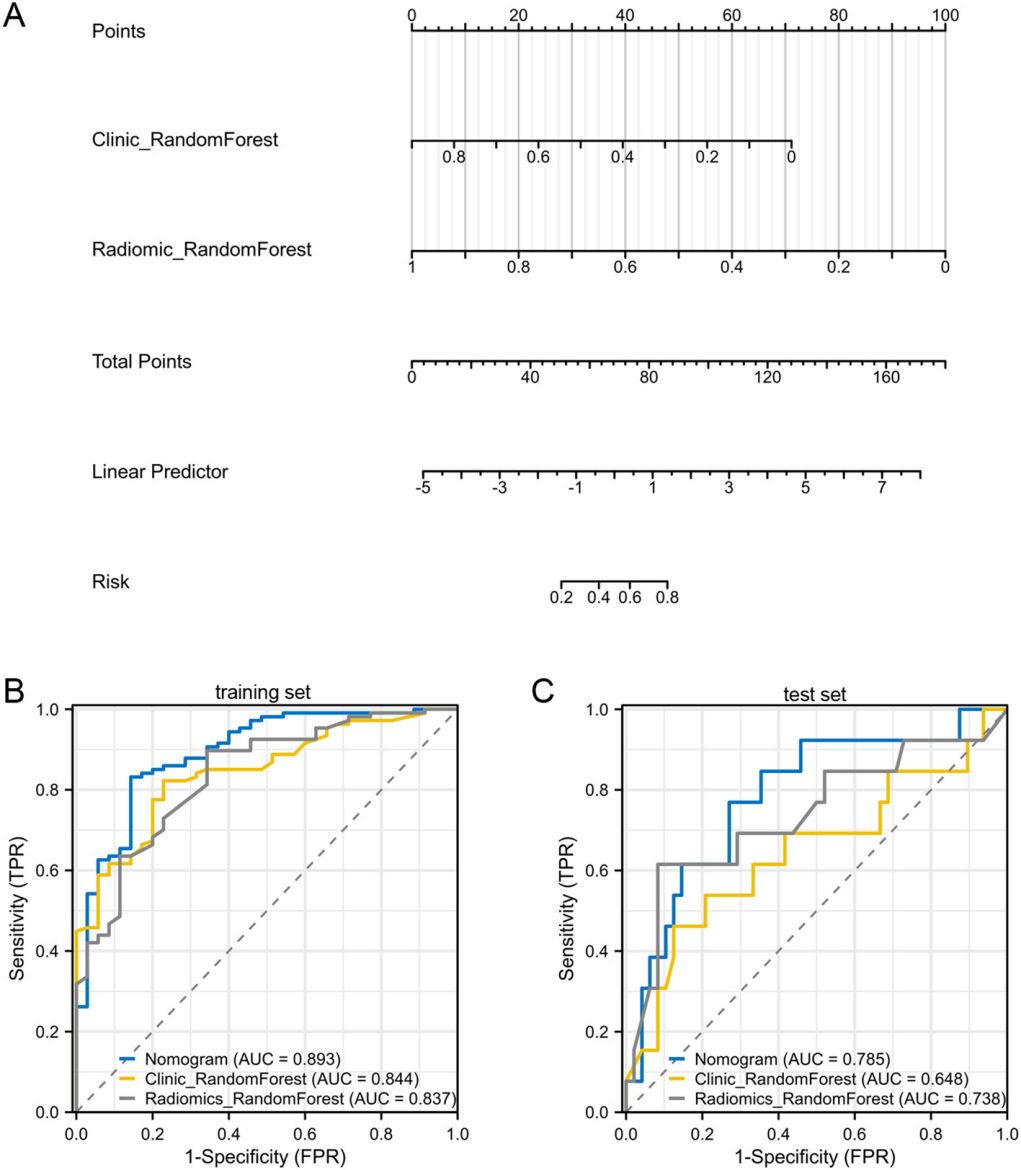
Assessment of a nomogram based on conventional and Hessian matrix-based radiomic model and clinicopatholigic model. **(A)** A nomogram based on the radiomic model and clinicopatholigic model. Compute the total score by summing the scores corresponding to “Clinic_RandomForest” and “Radiomic_RandomForest”. Reference the “Risk” scale with the calculated “Total Points” to determine the predicted probability of NAC efficacy. **(B)** ROC curves of clinicopatholigic model, radiomic model, and nomogram in training group. **(C)** ROC curves of clinicopatholigic model, radiomic model, and nomogram in test set.

To develop a more reliable prediction tool for assessing the efficacy of NAC in HER2-low breast cancer patients, a nomogram combining the best-performing machine learning models was established based on clinicopathological and radiomic signatures. Notably, the Random Forest model demonstrated the best performance in both clinical and radiomic analyses, making it the optimal choice for inclusion in the nomogram (Figure 6A).

To illustrate the application of the nomogram, two patients were used as examples. For Patient A, a premenopausal woman with cT2N1M0, ER (70%+), PR (70%+), HER2 (1+), and Ki67 20%, the clinicopathological signature was 0.48, which corresponded to 32 points. The radiomics signature was 0.12, corresponding to 90 points. This resulted in a total score of 112 points, which yielded a linear predictor of 2.5, and a predicted NAC efficacy probability of 0.12. After NAC, Patient A achieved Miller-Payne grade 2, indicating a poor response to NAC. For Patient B, a premenopausal woman with cT2N2M0, ER (90%+), PR (70%+), Ki67 80%, HER2 (2+), the clinicopathological signature was 0.71, corresponding to 15 points, and the radiomics signature was 0.72, corresponding to 50 points. The total score was 65 points, leading to a linear predictor of −1.99, and a predicted NAC efficacy probability of 0.97. Following NAC, Patient B achieved Miller-Payne grade 5.

When evaluated in the training set, the nomogram showed an accuracy of 0.84, with an AUC of 0.89 (Figure 6B). The sensitivity was 0.86, and the specificity was 0.83. In the validation cohort, the nomogram demonstrated good performance, with an AUC of 0.79 (Figure 6C). It achieved a sensitivity of 0.77 and a specificity of 0.73, suggesting that the nomogram could effectively identify both responders and non-responders to NAC.

**Figure 7:**
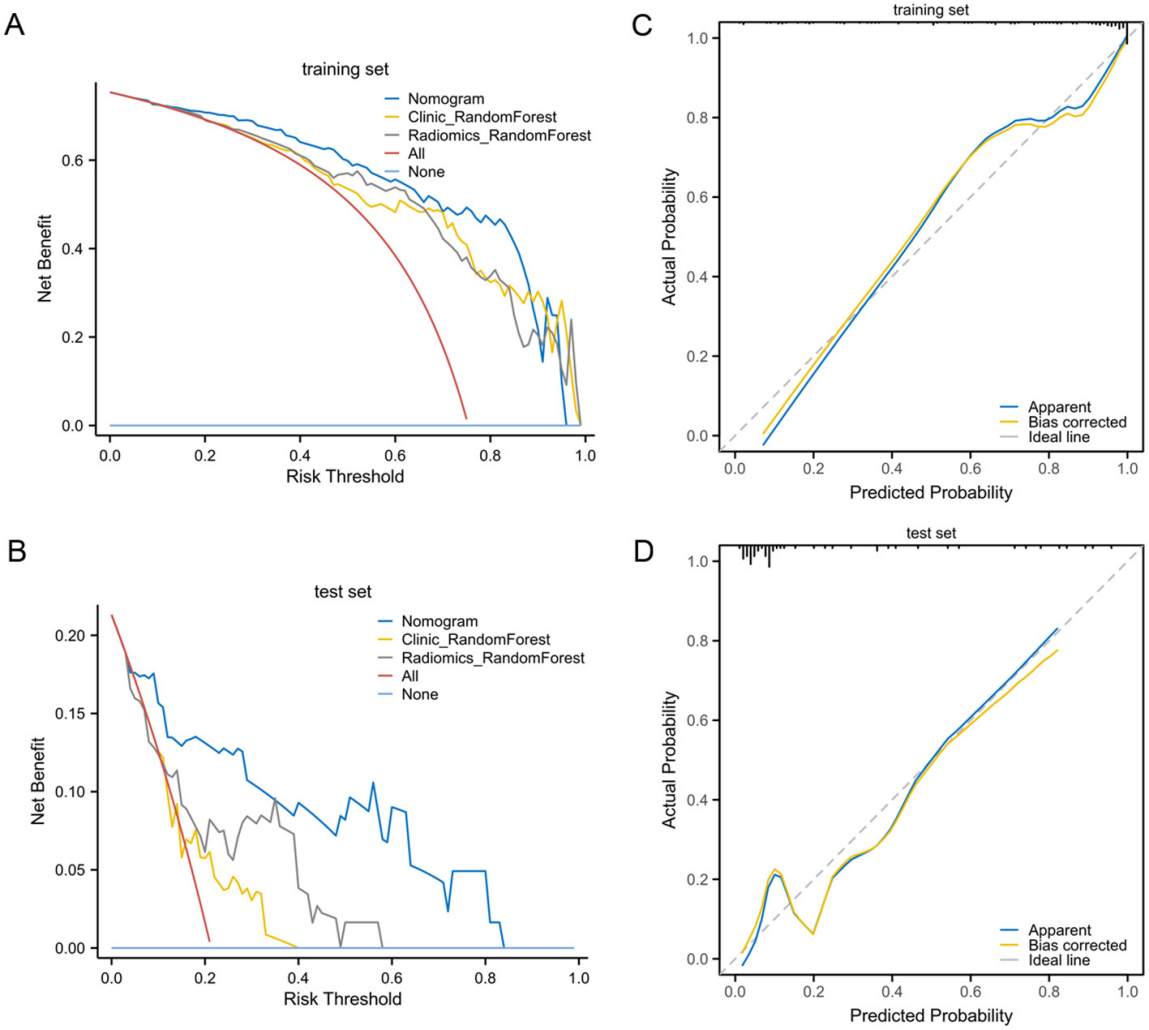
The calibration and DCA curves of radiomic model and clinicopatholigic model. **(A)** DCA evaluating the clinical usefulness in the training set; **(B)** DCA evaluating the clinical usefulness in the test set; **(C)** Calibration curve of nomogram in the training set; **(D)** Calibration curve of nomogram in the test set.

The DCA curves indicated that the nomogram provided greater net clinical benefit compared to both the radiomic model and the clinical model alone, suggesting that the nomogram offers a more robust tool for predicting NAC outcomes in HER2-low breast cancer patients (Figures 7A, B). The calibration plot demonstrated nomogram effectively distinguished between patients with varying NAC efficacy (Figures 7C, D).

## Discussion

This study aimed to construct a predictive model for evaluating the efficacy of NAC in HER2-low breast cancer patients. By integrating clinical factors with radiomic features, we demonstrated a promising approach for pinpointing patients expected to exhibit a positive response to NAC. The nomogram combining clinical and radiomic signatures outperformed traditional clinical models in terms of accuracy, specificity, and NPV. This work highlights the potential to improve treatment outcomes by providing a more precise and individualized therapeutic approach for HER2-low patients undergoing NAC.

A notable and important finding of this study is that, despite increasing attention on the efficacy of NAC for HER2-low breast cancer, much of the existing research has focused on late-stage patients, with limited studies on the efficacy of NAC during the neoadjuvant phase. Most available models for predicting chemotherapy response are based on clinicopathological factors, such as age, tumor size, hormone receptor status, and Ki-67 index. For instance, Liu et al. (13) found that Ki-67 expression, pathological grading, and changes in HER2 status after NAC were critical factors influencing the response to NAC in HER2-low breast cancer patients. Another study by Tang et al. (12) highlighted the significant role of lymph node staging and ER status in predicting NAC outcomes. While these clinical factors provide valuable insights, their predictive power can be limited, especially in HER2-low patients (11, 24).

HER2-low breast cancer is now understood to exhibit distinct characteristics that differentiate it from other subtypes, such as Luminal or TNBC. Research indicates that the pCR rate in HER2-low patients following NAC is notably lower than in other subtypes, with retrospective studies reporting pCR rates ranging from only 11.8%−16.4% (10–12). This low pCR rate emphasizes the need for a better predictive tool to tailor treatment strategies for these patients. By integrating radiomic features, this study provides a step forward in predicting NAC efficacy for HER2-low patients, allowing for more personalized treatment. Using advanced tools such as novel anti-HER2 ADCs may be an option for these patients, especially when more accurate prediction models are available to guide therapy selection.

In recent years, numerous studies have explored the application of radiomics models to predict NAC efficacy in breast cancer patients. Huang et al. (16) developed ensemble learning models using longitudinal multiparametric MRI to predict pCR in breast cancer. Their study enrolled 1,262 breast cancer patients from four centers, focusing on constructing a machine learning model for NAC prediction based on molecular subtypes. A stacking model was developed, which integrated pre-treatment, post-treatment, and delta-models, and demonstrated excellent performance. Similarly, Bitencourt et al. (25) focused on HER2-positive breast cancer patients, aiming to utilize radiomics models to predict the efficacy of NAC in this specific group. Their study incorporated both clinical and radiomics MRI features, resulting in machine learning models that achieved a sensitivity of 0.87, specificity of 0.80, and diagnostic accuracy of 0.84 in the validation set. Taken together, these results demonstrate the effectiveness of radiomics in predicting NAC outcomes in breast cancer patients, suggesting that radiomic features from MRI can significantly enhance treatment response predictions and guide clinical decision-making. However, a notable gap in the current literature is the limited exploration into the development of radiomics models for HER2-low patients.

A key innovation in this study is the incorporation of Hessian matrix-based radiomic features, which offer distinct advantages over conventional radiomic features. The Hessian matrix approach is particularly valuable for capturing fine-grained texture information that reflects the tumor microenvironment's complexity (20). It can detect subtle changes in tumor morphology and vascularity, which might not be visible in traditional imaging or clinical assessments (26). While the application of the Hessian matrix to radiomics features is a promising approach, it remains underexplored in the context of predicting the efficacy of NAC for HER2-low breast cancer patients. However, most radiomic features incorporated into the model are traditional radiomic features. These features, derived from well-established techniques in radiomics, possess distinct characteristics that can offer specific advantages during NAC efficacy prediction in breast cancer. The original_shape_Elongation feature measures the extent to which the tumor's shape deviates from spherical symmetry, which can reflect the tumor's growth pattern and the presence of irregularities often associated with aggressive or less responsive cancers (27, 28). Wavelet-based features capture texture information at multiple scales, enabling the detection of heterogeneity within the tumor (29). The wavelet_LLL_ngtdm_Coarseness feature, derived from NGTDM, evaluates the coarseness or smoothness of the texture within the tumor (30). Incorporating these traditional radiomic features into the model provides several advantages over clinical and pathological features alone. Radiomic features offer a more granular analysis of tumor heterogeneity, which is critical for accurately predicting chemotherapy response, particularly in the case of HER2-low breast cancer.

However, several limitations to this study should be acknowledged. One major limitation is the retrospective design, which may introduce bias due to patient selection and variations in treatment protocols. Besides, our study lacks external validation due to the limited number of cohorts meeting our specific inclusion criteria. As detailed in the Methods, strict eligibility requirements—including HER2-low breast cancer, receipt of systemic chemotherapy prior to surgery, and availability of pre-NAC DCE-MRI scans—have significantly limited potential external cohorts. While a small number of candidate cohorts were identified, most were excluded due to lack of pre-NAC MR examinations. The remaining had a small sample size (*n* < 20) to support meaningful validation. In the future, independent external validation data will be needed to validate the model. Specifically, we will collaborate with 2–3 regional tertiary hospitals with standardized breast cancer data management systems. Our goal is to enroll 300–500 HER2-low breast cancer patients prospectively, ensuring consistent DCE-MRI acquisition protocols and clinical data collection standards across all centers. This multi-center cohort will be used to externally validate and optimize our radiomic model, thereby enhancing its reliability and applicability in broader clinical settings. Moreover, while the Random Forest model demonstrated a good performance in this study, the interpretation of its decision-making process remains a challenge, as it operates as a “black-box” model, which limits its clinical applicability for some practitioners who may prefer more interpretable models.

Future studies should aim to validate the nomogram in larger, multi-center cohorts to ensure its robustness and generalizability across diverse patient populations. In addition, future research could explore combining radiomic features with molecular biomarkers, such as genetic mutations or protein expression profiles, to refine the predictive model. It would also be valuable to investigate the use of real-time imaging data, such as dynamic contrast-enhanced MRI or PET scans, during the treatment course to monitor tumor response dynamically and adjust treatment plans accordingly.

Overall, this study provides new insights into predicting NAC efficacy in HER2 low-expressing breast cancer patients and suggests that integrating advanced imaging techniques like radiomics and the Hessian matrix could enhance treatment prediction models. By incorporating these technologies, it may be possible to better identify patients who would benefit from intensified chemotherapy or more novel targeted therapies. Ultimately, this could lead to more personalized treatment strategies, improving outcomes for a challenging group of patients. Subsequent research should investigate the practical applications of these models and evaluate their performance in actual clinical environments to validate their efficacy.

## Data Availability

The original contributions presented in the study are included in the article/Supplementary material, further inquiries can be directed to the corresponding author.

## References

[1] Bray F Laversanne M Sung H Ferlay J Siegel RL Soerjomataram I . Global cancer statistics 2022: GLOBOCAN estimates of incidence and mortality worldwide for 36 cancers in 185 countries. CA-Cancer J Clin. (2024) 74:229–63. doi: 10.3322/caac.21834 38572751

[2] Cheng X. A comprehensive review of HER2 in cancer biology and therapeutics. Genes. (2024) 15:903. doi: 10.20944/preprints202406.0515.v1 39062682 PMC11275319

[3] Piccart M Procter M Fumagalli D de Azambuja E Clark E Ewer MS . Adjuvant pertuzumab and trastuzumab in early HER2-positive breast cancer in the APHINITY trial: 6 years' follow-up. J Clin Oncol. (2021) 39:1448–57. doi: 10.1200/JCO.20.01204 33539215

[4] Swain SM Miles D Kim SB Im YH Im SA Semiglazov V . Pertuzumab, trastuzumab, and docetaxel for HER2-positive metastatic breast cancer (CLEOPATRA): end-of-study results from a double-blind, randomised, placebo-controlled, phase 3 study. Lancet Oncol. (2020) 21:519–30. doi: 10.1016/S1470-2045(19)30863-0 32171426

[5] Bardia A Hu X Dent R Yonemori K Barrios CH O'Shaughnessy JA . Trastuzumab deruxtecan after endocrine therapy in metastatic breast cancer. New Engl J Med. (2024) 391:2110–22. doi: 10.1056/NEJMoa2407086 39282896

[6] Mosele F Deluche E Lusque A Le Bescond L Filleron T Pradat Y . Trastuzumab deruxtecan in metastatic breast cancer with variable HER2 expression: the phase 2 DAISY trial. Nat Med. (2023) 29:2110–20. doi: 10.1038/s41591-023-02478-2 37488289 PMC10427426

[7] Huober J von Minckwitz G. Neoadjuvant therapy - what have we achieved in the last 20 years? Breast Care. (2011) 6:419–26. doi: 10.1159/000335347 22419894 PMC3290030

[8] Navarro-Cecilia J Duenas-Rodriguez B Luque-Lopez C Ramirez-Exposito MJ Martinez-Ferrol J Ruiz-Mateas A . Intraoperative sentinel node biopsy by one-step nucleic acid amplification (OSNA) avoids axillary lymphadenectomy in women with breast cancer treated with neoadjuvant chemotherapy. Eur J Surg Onc. (2013) 39:873–9. doi: 10.1016/j.ejso.2013.05.002 23711734

[9] Wolf DM Yau C Wulfkuhle J Brown-Swigart L Gallagher RI Lee P . Redefining breast cancer subtypes to guide treatment prioritization and maximize response: predictive biomarkers across 10 cancer therapies. Cancer Cell. (2022) 40:609–23. doi: 10.1016/j.ccell.2022.05.005 35623341 PMC9426306

[10] Zhao S Wang Y Zhou A Liu X Zhang Y Zhang J. Neoadjuvant chemotherapy efficacy and prognosis in HER2-low and HER2-zero breast cancer patients by HR status: a retrospective study in China. PeerJ. (2024) 12:e17492. doi: 10.7717/peerj.17492 38827304 PMC11143972

[11] Guan F Ju X Chen L Ren J Ke X Luo B . Comparison of clinicopathological characteristics, efficacy of neoadjuvant therapy, and prognosis in HER2-low and HER2-ultralow breast cancer. Diagn Pathol. (2024) 19:131. doi: 10.1186/s13000-024-01557-3 39350260 PMC11441256

[12] Tang L Li Z Jiang L Shu X Xu Y Liu S. Efficacy evaluation of neoadjuvant chemotherapy in patients with HER2-low expression breast cancer: a real-world retrospective study. Front Oncol. (2022) 12:999716. doi: 10.3389/fonc.2022.999716 36605428 PMC9810386

[13] Liu JJ Zhang Y Zhang SC Liu X Wang SN Liu XY . Analysis of factors influencing the efficacy of NAC and prognosis between HER2-zero and HER2-low HR negative breast cancer. Front Cell Dev Biol. (2024) 12:1417271. doi: 10.3389/fcell.2024.1417271 39650721 PMC11621093

[14] Aerts HJWL Velazquez ER Leijenaar RTH Parmar C Grossmann P Carvalho S . Decoding tumour phenotype by noninvasive imaging using a quantitative radiomics approach. Nat Commun. (2014) 5:4006. doi: 10.1038/ncomms5644 24892406 PMC4059926

[15] Gillies RJ Kinahan PE Hricak H. Radiomics: images are more than pictures, they are data. Radiology. (2016) 278:563–77. doi: 10.1148/radiol.2015151169 26579733 PMC4734157

[16] Huang Y Zhu T Zhang X Li W Zheng X Cheng M . Longitudinal MRI-based fusion novel model predicts pathological complete response in breast cancer treated with neoadjuvant chemotherapy: a multicenter, retrospective study. EClinicalMedicine. (2023) 58:101899. doi: 10.1016/j.eclinm.2023.101899 37007742 PMC10050775

[17] Yu Y Wang Z Wang Q Su X Li Z Wang R . Radiomic model based on magnetic resonance imaging for predicting pathological complete response after neoadjuvant chemotherapy in breast cancer patients. Front Oncol. (2023) 13:1249339. doi: 10.3389/fonc.2023.1249339 38357424 PMC10865896

[18] Zeng Q Ke M Zhong L Zhou Y Zhu X He C . Radiomics based on dynamic contrast-enhanced MRI to early predict pathologic complete response in breast cancer patients treated with neoadjuvant therapy. Acad Radiol. (2023) 30:1638–47. doi: 10.1016/j.acra.2022.11.006 36564256

[19] Fusco R Sansone M Filice S Petrillo A. Breast contrast-enhanced MR imaging: semiautomatic detection of vascular map. Breast Cancer. (2016) 23:266–72. doi: 10.1007/s12282-014-0565-8 25239166

[20] Xie T Gong J Zhao Q Wu C Wu S Peng W . Development and validation of peritumoral vascular and intratumoral radiomics to predict pathologic complete responses to neoadjuvant chemotherapy in patients with triple-negative breast cancer. BMC Med Imaging. (2024) 24:136. doi: 10.1186/s12880-024-01311-7 38844842 PMC11155097

[21] Zhang B Yu Y Mao Y Wang H Lv M Su X . Development of MRI-based deep learning signature for prediction of axillary response after NAC in breast cancer. Acad Radiol. (2024) 31:800–11. doi: 10.1016/j.acra.2023.10.004 37914627

[22] Tustison NJ Avants BB Cook PA Zheng Y Egan A Yushkevich PA . N4ITK: improved N3 bias correction. In: IEEE T Med Imaging, Vol. 29. Piscataway, NJ: IEEE (2010). p. 1310–20. doi: 10.1109/TMI.2010.2046908 20378467 PMC3071855

[23] Lundberg SM Erion GG Lee S. Consistent individualized feature attribution for tree ensembles. arXiv. [preprint]. (2018). arXiv:1802.03888. doi: 10.48550/arXiv.1802.03888

[24] Garufi G Mastrantoni L Maliziola N Monte ED Arcuri G Frescura V . Activity and efficacy of neoadjuvant chemotherapy in luminal-HER2 negative early breast cancer according to HER2 score (Low vs Score 0): a retrospective study. Clin Breast Cancer. (2025) 25:431–43. doi: 10.2139/ssrn.5047426 40155250

[25] Bitencourt A Gibbs P Rossi SC Daimiel I Lo GR Fox MJ . MRI-based machine learning radiomics can predict HER2 expression level and pathologic response after neoadjuvant therapy in HER2 overexpressing breast cancer. EBioMedicine. (2020) 61:103042. doi: 10.1016/j.ebiom.2020.103042 33039708 PMC7648120

[26] Le QC Arimura H Ninomiya K Kabata Y. Radiomic features based on Hessian index for prediction of prognosis in head-and-neck cancer patients. Sci Rep. (2020) 10:21301. doi: 10.1038/s41598-020-78338-7 33277570 PMC7718925

[27] Pasquali S Iadecola S Vanzulli A Infante G Bologna M Corino V . Radiomic features of primary retroperitoneal sarcomas: a prognostic study. Eur J Cancer. (2024) 213:115120. doi: 10.1016/j.ejca.2024.115120 39541785

[28] He M Singh R Wang M Ho G Wong EMF Chiu KWH . CT-based radiomics model to predict platinum sensitivity in epithelial ovarian carcinoma: a multicentre study. Cancer Imaging. (2025) 25:85. doi: 10.1186/s40644-025-00906-9 40611334 PMC12225207

[29] Mathews C Mohamed A. Deep classification of glioma grade using 3D wavelet features. In: 2022 International Conference for Advancement in Technology (ICONAT): 2022/1/1 2022. Goa, India: IEEE (2022). p. 1–5. doi: 10.1109/ICONAT53423.2022.9725929

[30] Chaddad A Daniel P Niazi T. Radiomics evaluation of histological heterogeneity using multiscale textures derived from 3D wavelet transformation of multispectral images. Front Oncol. (2018) 8:96. doi: 10.3389/fonc.2018.00096 29670857 PMC5893871

