# Supplementary material for: A radiomic model leveraging conventional and Hessian matrix-based radiomic features from DCE-MRI for predicting efficacy of neoadjuvant chemotherapy in patients with HER2-low breast cancer
Source: Front Med (Lausanne). 2026 Jan 13;12:1639977. doi: 10.3389/fmed.2025.1639977 (PMC12835305; doi:10.3389/fmed.2025.1639977)
Supplement: Supplementary file 1 [file Table_1.docx]

Supplementary Table 1. Stratified comparison of clinical characteristics between training and validation Cohorts

|  |  |  | Training cohort | Validation cohort | P value |
| --- | --- | --- | --- | --- | --- |
| Age | Responders | | 45.80 ± 8.27 | 49.31 ± 8.35 | 0.20 |
|  | Non-responders | | 51.64 ± 10.00 | 52.89 ± 9.59 | 0.46 |
| Menopausal status | Responders | Premenopause | 28(80.00%) | 8 (61.54%) | 0.15 |
|  |  | Post-menopause | 7 (20.00%) | 5 (38.46%) |  |
|  | Non-responders | Premenopause | 51 (47.66%) | 22 (45.83%) | 0.60 |
|  |  | Post-menopause | 56 52.34%) | 26 (54.17%) |  |
| ER status | Responders | Positive | 30 (85.71%) | 11 (84.62%) | 0.77 |
|  |  | Negative | 5 (14.29%) | 2 (15.38%) |  |
|  | Non-responders | Positive | 103 (96.26%) | 43 (89.58%) | 0.20 |
|  |  | Negative | 4 (3.74%) | 5 (10.42%) |  |
| PR status | Responders | Positive | 22 (62.86%) | 8 (61.54%) | 0.98 |
|  |  | Negative | 13 (37.14%) | 5 (38.46%) |  |
|  | Non-responders | Positive | 87 (81.31%) | 41 (85.42%) | 0.69 |
|  |  | Negative | 20 (18.69%) | 7 (14.58%) |  |
| Her-2 status | Responders | IHC 1+ | 28(80.00%) | 8 (61.54%) | 0.35 |
|  |  | IHC 2+/FISH- | 7 (20.00%) | 5 (38.46%) |  |
|  | Non-responders | IHC 1+ | 75 (70.09%) | 36 (75.00%) | 0.66 |
|  |  | IHC 2+/FISH- | 32 (29.91%) | 12 (25.00%) |  |
| Ki-67 index | Responders | | 50 (30, 65) | 30 (30, 50) | 0.40 |
|  | Non-responders | | 30 (20, 40) | 30 (18.75, 50) | 0.97 |
| cT | Responders | T1 | 1 (2.86%) | 3 (23.08%) | 0.17 |
|  |  | T2 | 21 (60.00%) | 5 (38.46%) |  |
|  |  | T3 | 11 (31.43%) | 5 (38.46%) |  |
|  |  | T4 | 2 (5.71%) | 0 (0.00%) |  |
|  | Non-responders | T1 | 11 (10.28%) | 5 (10.42%) | 0.65 |
|  |  | T2 | 50 (46.73%) | 23 (47.92%) |  |
|  |  | T3 | 37 (34.58%) | 14 (29.17%) |  |
|  |  | T4 | 9 (8.41%) | 6 (12.50%) |  |
| cN | Responders | N0 | 4 (11.43%) | 0 (0.00%) | 0.55 |
|  |  | N1 | 28 (80.00%) | 12 (92.31%) |  |
|  |  | N2 | 2 (5.71%) | 1 (7.69%) |  |
|  |  | N3 | 1 (2.86%) | 0 (0.00%) |  |
|  | Non-responders | N0 | 8 (7.48%) | 5 (10.42%) | 0.44 |
|  |  | N1 | 83 (77.57%) | 34 (70.83%) |  |
|  |  | N2 | 10 (9.35%) | 5 (10.42%) |  |
|  |  | N3 | 6 (5.61%) | 4 (8.33%) |  |
| ER value | Responders | | 70 (4, 90) | 80 (5, 90) | 0.92 |
|  | Non-responders | | 80 (70, 90) | 90 (70, 90) | 0.29 |
| PR value | Responders | | 30 (0, 70) | 10 (0, 30) | 0.56 |
|  | Non-responders | | 40 (5, 85) | 60 (10, 90) | 0.29 |
| TILs | Responders | | 5 (3, 10) | 10 (8.25, 12.5) | 0.49 |
|  | Non-responders | | 5 (3, 10) | 5 (5, 10) | 0.56 |
